# Larger infarct size but equal protection by ischemic conditioning in septum and anterior free wall of pigs with LAD occlusion

**DOI:** 10.14814/phy2.14236

**Published:** 2019-10-09

**Authors:** Andreas Skyschally, Helene Hagelschuer, Petra Kleinbongard, Gerd Heusch

**Affiliations:** ^1^ Institute for Pathophysiology West German Heart and Vascular Center University of Essen Medical School Essen Germany

**Keywords:** Infarct size, ischemia, ischemic conditioning, myocardial infarction, pig, reperfusion

## Abstract

The ischemic area at risk (AAR) is one major determinant of infarct size (IS). In patients, the largest AAR is seen with a proximal occlusion of the left anterior descending (LAD) coronary artery, which serves parts of the septum and of the anterior free wall. It is not clear, whether regional differences in the perfusion territories also impact on IS and the magnitude of cardioprotection by ischemic conditioning. We have retrospectively analyzed 132 experiments in pigs, which have a similar LAD perfusion territory as humans. The LAD was occluded for 60 min with subsequent 180 min reperfusion. Cardioprotection by either local ischemic pre‐ or postconditioning or remote ischemic pre‐ or perconditioning was induced in 93 pigs. The AAR was demarcated by blue dye staining, and IS was assessed by triphenyltetrazolium chloride (TTC) staining. Using digital planimetry, the AAR was separated into sections unequivocally located in the septum (AAR_S_) or the anterior free wall (AAR_AFW_). Relative IS was calculated for AAR_S_ or AAR_AFW_. AAR_AFW_ was larger than AAR_S_ (51 ± 9% vs. 34 ± 8% of total AAR; mean ± SD, *P* < 0.001). Regional myocardial blood flow (microspheres) was not different between septum and anterior free wall. IS without ischemic conditioning tended to be larger in AAR_S_ than in AAR_AFW_ (50 ± 17% vs. 44 ± 19%; % of AAR_AWF_ or AAR_S_, respectively; *P* = 0.075). Also, with robust IS reduction by ischemic conditioning, the difference in relative IS remained (AAR_S_: 27 ± 16%; AAR_AFW_: 21 ± 16%; *P* = 0.01). There is a somewhat greater susceptibility for infarction in septal than anterior free wall myocardium. However, ischemic conditioning still reduces IS in both septal and anterior free wall myocardium.

## Introduction

In patients surviving an acute myocardial infarction, infarct size (IS) is a major determinant of prognosis (Stone et al. 2016; Heusch and Gersh, 2017). IS depends on the amount of myocardium at risk, the duration of ischemia, the residual blood flow to the myocardium at risk during ischemia, and somewhat on the systemic hemodynamics during ischemia. Whether regional myocardial mechanics and metabolism also impact on final IS is less clear. Ischemic conditioning maneuvers attenuate IS (Hausenloy et al. 2016), and such protection can be recruited by cycles of brief, nonlethal ischemia/reperfusion either locally in the heart or remotely from tissues other than the heart. Cardioprotection by ischemic conditioning can be induced prior to ischemia as local ischemic preconditioning (IPC) (Murry et al. 1986) or as remote ischemic preconditioning (RIPC) (Przyklenk et al. 1993; Heusch et al. 2015), during ischemia as remote ischemic perconditioning (RPER) (Schmidt et al. 2007; Kleinbongard et al. 2018), and locally at the onset of reperfusion as ischemic postconditioning (POCO) (Zhao et al. 2003).

The cause of acute myocardial infarction is usually the thrombotic occlusion of a large epicardial coronary artery after rupture of an atherosclerotic plaque, and the site of such thrombotic occlusion largely determines the area at risk (AAR). The perfusion territories of the three major coronary vessels differ in their size (Mahmarian et al. 1991), and the amount of myocardium at risk increases with more proximal occlusion sites. The left anterior descending (LAD) coronary artery perfusion territory is the largest in the human heart (Mahmarian et al. 1991), and its proximal occlusion puts the anterior part of the septum and the anterior ventricular free wall at risk. Of note, the consequences of myocardial infarction in these areas differ. Septal infarction bears the risk of septal wall rupture, a rare but nevertheless severe complication (Pohjola‐Sintonen et al. 1989). Due to its anatomical proximity of the conduction system, septal infarction more frequently results in a left bundle branch block with worse clinical outcome (Erne et al. 2017). On the other hand, infarction of the anterior free wall is associated with reduced left ventricular function due to the high radial strain in anterior and apical regions of the left ventricle under normal conditions (Bogaert and Rademakers, 2001). In anesthetized open‐chest pigs subjected to severe hypoperfusion and subsequent reperfusion of the LAD perfusion territory, Schulz et al. reported a relatively larger IS, when normalized to the respective AAR, in the septum than in the anterior free wall (Schulz et al. 2005), suggesting a higher susceptibility of septal myocardium for infarction. What are the underlying reasons for such different susceptibility to infarction? The distribution of mechanical load in the septum is different from that in the anterior free wall. The outer myocardial layers of the septum are the inner layers of the right ventricle, and fiber strain in the outer layers of the septum exceeds that in the inner layers. Radial strain in the anterior free wall is higher than that in the septum (Bogaert and Rademakers, 2001). However, peak systolic wall stress determines myocardial oxygen consumption and is similar in the septum and anterior wall (Balzer et al. 1999). Potential mechanical differences may also impact on extravascular compression and regional myocardial blood flow, facilitating the redistribution of blood flow between left and right ventricular perfusion territories of the LAD (“right ventricular steal phenomenon”) during hypoperfusion (Guth et al. 1991). In humans, resting myocardial blood flow is slightly higher in the anterior wall than the septum (Gropler et al. 1990; Chareonthaitawee et al. 2001), rendering it potentially more susceptible to myocardial infarction, as seen in baboons (Ghaleh et al. 1996). There is no apparent difference in myocardial oxidative or glucose metabolism between the septal myocardium and the anterior wall (Hicks et al. 1991). However, there appears to be a chamber‐specific difference in right and left heart handling of reactive oxygen species (Schlüter et al. 2018), possibly also relevant to septal myocardium and its susceptibility to infarction.

There is little information on regional differences in the efficacy of cardioprotection by ischemic conditioning. In pigs, IS reduction by local ischemic preconditioning is more pronounced in the septum; the larger IS in the septum than the anterior free wall is no longer seen after ischemic preconditioning (Schulz et al. 2005). Whether such spatial differences in the magnitude of cardioprotection also exist for more clinically relevant ischemic conditioning maneuvers such as ischemic postconditioning (Zhao et al. 2003), or remote ischemic conditioning prior to (Heusch et al. 2015) or during ischemia (Schmidt et al. 2007) is currently not known. Cardioprotection by remote ischemic conditioning involves vagal nerves (Lieder et al. 2018; Basalay et al. 2018). Therefore, regional differences in cardiac innervation might impact on the magnitude of cardioprotection in septal and anterior free wall compartments. Thick vagal fibers are histologically mainly seen in human epicardium and subepicardium, whereas thin fibers are seen in subendocardial layers. Vagal fiber density is higher in right than left ventricular myocardium (Kawano et al. 2003). Such gradient of vagal nerve density between right and left ventricular myocardium is also seen in pigs (Crick et al. 1999). Sympathetic nerve fibers travel below the epicardium along the large epicardial vessels and innervate the anterior free wall (Geis and Kaye, 1968; Heusch et al. 1987). However, the role of sympathetic nerves in cardioprotection by ischemic conditioning is less clear (Kleinbongard et al. 2017).

We have now retrospectively analyzed experiments in anesthetized, open‐chest pigs subjected to LAD occlusion and reperfusion. The pig heart closely resembles the human heart in its anatomy, hemodynamics, and its LAD perfusion territory (Heusch et al. 2011). We determined AAR and IS in the septum and anterior free wall after proximal LAD occlusion. In addition, we analyzed and compared the magnitude of cardioprotection by local ischemic preconditioning (Gent et al. 2017), ischemic postconditioning (Skyschally et al. 2013), remote ischemic preconditioning (Skyschally et al. 2015), and remote ischemic perconditioning (Kleinbongard et al. 2018; Skyschally et al. 2018) in the septum and anterior free wall.

## Material and methods

The experimental protocols were approved by the Bioethical Committee of the district of Düsseldorf (G1018/09, G1163/10, G1240/11, G1259/11, G1320/12, G1407/14, G1652/17, and G1655/18). Unless otherwise indicated, materials were obtained from Sigma‐Aldrich (Deisenhofen, Germany).

### Experimental preparation

The experiments were performed between November 2012 and November 2018, and their results have already been reported in a different context (Skyschally et al. 2013; Skyschally et al. 2015; Gent et al. 2017; Kleinbongard et al. 2018; Skyschally et al. 2018; Lieder et al. 2018). All experiments were performed in healthy Göttingen minipigs (male, body weight: ~30 kg, age: 12–14 months; Ellegaard, Dalmose, Denmark). The pigs had free access to water and were kept in tiled rooms (~2 m^2^/pig) with straw‐bedding, at 12 h/12 h light/dark cycles. Only male pigs were used, as sex‐based differences were not subject of our studies. The pigs were not subjected to any medication other than anesthesia prior to or during the experiment. Pigs were sedated with flunitrazepam (0.4 mg/kg i.m., Sigma‐Aldrich, Munich, Germany). Anesthesia was induced by etomidate (0.3 mg/kg, i.v. Hypnomidat; Janssen‐Cilag, Neuss, Germany) and sufentanil (1 µg/kg, i.v. Sufentanil‐hameln; hameln pharma plus gmbh, Hameln, Germany). After tracheotomy, pigs were mechanically ventilated with oxygen‐enriched air supplemented with isoflurane (2%, Forene, AbbVie, Ludwigshafen, Germany) to maintain anesthesia. The pigs were placed on a heated table and covered with drapes to keep core body temperature, as measured by an esophageal probe, between 36 and 38°C. The left jugular vein was cannulated for volume replacement, and the right common carotid artery was cannulated to measure arterial pressure. Arterial blood gases and glucose levels were regularly monitored and kept within the physiological range. The heart was exposed through a left lateral thoracotomy and instrumented with a micromanometer (Codan‐PVB, Lensahn, Germany) in the left ventricle. A Teflon catheter in the left atrium was used for injection of fluorescent microspheres to measure regional transmural myocardial blood flow (TMBF). A Teflon catheter, placed in the descending thoracic aorta, was used to withdraw blood as reference for the regional blood flow measurement (Kowallik et al. 1991). A silicon‐coated suture was placed around the LAD distal to its second diagonal branch for later coronary occlusion. Ventricular fibrillation was immediately terminated by internal defibrillation (Skyschally et al. 2017).

### Protocols

Ischemia/reperfusion (I/R; *n* = 39). The suture around the LAD was carefully tightened against a soft silicone plate. After 60‐min coronary occlusion, reperfusion was induced by quick release and removal of the suture and confirmed by the disappearance of the light blue color and the reappearance of red color on the surface of the reperfused myocardium. Reperfusion was continued for 180 min.

Ischemic preconditioning + I/R (IPC + I/R; *n* = 16). Ischemic preconditioning was induced by two 3‐min LAD occlusions, separated by 2 min reperfusion. The index ischemia was started 15 min later. The subsequent protocol was identical to I/R.

I/R + ischemic postconditioning (I/R + POCO; *n* = 18). Ischemic postconditioning was induced by four cycles of 1‐min LAD occlusion followed by 1 min reperfusion each, starting at 1 min reperfusion. The subsequent protocol was identical to I/R.

Remote ischemic preconditioning + I/R (RIPC + I/R; *n* = 31). For remote ischemic preconditioning, a tourniquet was applied around the left hind limb. RIPC was induced by four cycles of 5 min occlusion followed by 5 min reperfusion, starting 40 min before thoracotomy. Skin cyanosis was taken to indicate leg ischemia and blushing to indicate reperfusion. The subsequent protocol was identical to I/R.

I/R + remote ischemic perconditioning (I/R + RPER; *n* = 28). Remote ischemic perconditioning was performed in the same way as RIPC, but was started 20 min after onset of ischemia. The subsequent protocol was identical to I/R.

### Systemic hemodynamics

Heart rate, maximal left ventricular pressure (LVP_max_), and maximal rate of rise of left ventricular pressure (dP/dt_max_) were averaged off‐line over a period of 10 consecutive cardiac cycles (CORDAT II) (Skyschally et al. 1993) at baseline, 5 and 55 min after the onset of ischemia, and at 10, 30, 60, and 120 min reperfusion. Intervals with premature beats and periods of ventricular tachycardia/fibrillation were excluded from analysis.

### ST‐segment elevation

ST‐segment elevation was measured, as previously described (Kleinbongard et al. 2018; Amanakis et al. 2019). In brief, a single‐channel surface ECG was continuously recorded using a calibrated (1 mV reference) amplifier. Due to the surgical preparation and use of a metal rib retractor, the recorded ECG‐lead appeared similar to a V2 Wilson lead in humans. ST‐segment elevation was defined as the amplitude difference between a point 30 ms before the *P* wave and a second point 20 ms after the J‐point (Cooper et al. 2002; Prasad et al. 2004). Analysis was performed off‐line using digital calipers (Labchart 8, AD Instruments Pty Ltd, New South Wales, Australia) at baseline, 5 and 55 min after onset of ischemia, and at 10, 30, 60, and 120 min reperfusion, averaging 30 consecutive cardiac cycles; premature beats or periods of ventricular tachycardia/fibrillation were excluded. Of note, the position of the animal remained the same throughout the course of ECG registration to avoid changes in ECG amplitude.

### Area at risk and infarct size

At 180 min reperfusion, the LAD was re‐occluded at the same location as for the index ischemia, and 5‐ml blue dye (Patentblau V, Guerbet, Sulzbach, Germany) was quickly injected into the left atrium to delineate the AAR as remaining unstained.

The pig was euthanized by rapid injection of 20 mL potassium chloride solution (2 mol/L), and the heart was quickly removed from the chest, rinsed with cold saline, and cut into five slices perpendicular to the ventricular long axis. Slices were documented using a digital camera, and the slice shape and the demarcated AAR were traced on a transparent film. Thereafter, infarcted tissue was demarcated by triphenyltetrazolium chloride (TTC) staining (1% TTC; Sigma‐Aldrich Chemie, dissolved in 90 mmol/L sodium phosphate buffer containing 8% dextran). The TTC‐stained slices were again photographed, and the tissue areas that remained unstained by TTC were transferred to the same transparent film that was used to document the AAR. Particular care was taken to proper realign the slices using landmarks, such as the position of papillary muscles. The transparent films were scanned and later analyzed.

**Figure 1:**
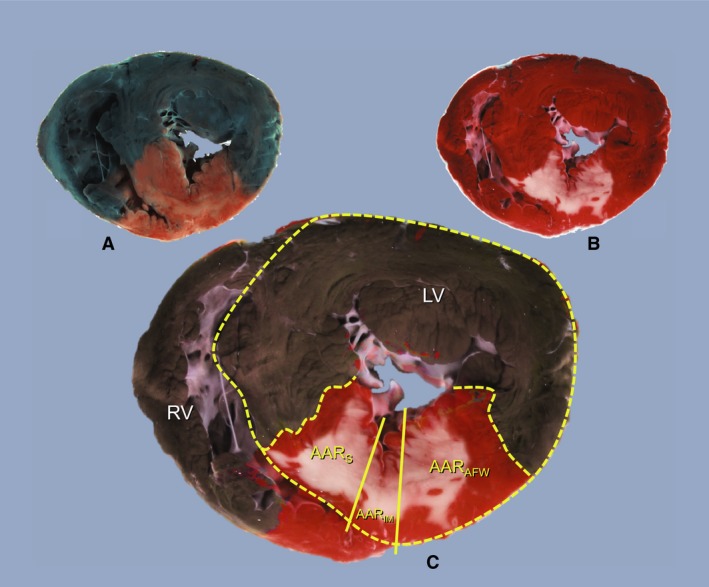
Display of a representative heart slice stained for demarcation of the area at risk (A) and infarcted myocardium (B). In the overlay (C), the yellow dashed lines mark the outer border of the left ventricle and the total area at risk, and the solid yellow lines separate the area at risk into a septal segment (AAR_S_), an intermediate segment (AAR_IM_), and a segment in the left ventricular anterior free wall (AAR_AFW_).

The retrospective allocation of AAR and infarcted tissue to either the septum or the free anterior wall, respectively, was performed blinded with regard to the treatment group. AAR and IS calculations were confined only to left ventricular myocardium. At the intersections of left and right ventricular myocardium, the outer circumference of the left ventricle was extrapolated to separate right from left ventricular myocardium. The total AAR was segmented into three areas (Fig. 1) using straight‐centered lines. AAR within the septum (AAR**_S_**) was defined as myocardium covered by left and right ventricular endocardium, AAR within the left ventricular anterior free wall (AAR_AFW_) was defined as myocardium covered by left ventricular endocardium and left ventricular epicardium. A small intermediate segment (AAR_IM_) located between AAR_S_ and AAR_AWF_ did not comply with these definitions was therefore not considered in further analyses and is only displayed for data completeness. The areas of left ventricular myocardium, total AAR, AAR_S_, AAR_AFW_, AAR_IM_, as well as the areas of infarcted myocardium within the total AAR and the three AAR segments, respectively, were measured by digital planimetry (Skaletti 2.5, Dr. F. Lechtenberg, Germany) and averaged for both sides of each slice. Using the slice weight for normalization, tissue masses for each compartment were calculated to express total AAR as fraction of the left ventricle. The relative size of AAR_S_, AAR_AFW_, and AAR_IM_ segments was referenced to the size of the total AAR. IS was calculated as fraction of the total AAR and separately within the three subsegments, respectively.

### Transmural myocardial blood flow

TMBF was measured using fluorescent microspheres (Kowallik et al. 1991). In brief, at baseline and at 5 min ischemia, respectively, 3–5 × 10^6^ microspheres of different colors (Fluospheres polystyrene 15 µm, Life technologies/Molecular probes, Darmstadt, Germany) were slowly injected into the left atrium. The microspheres mixed homogenously with the blood, distributed proportionally to tissue blood flow, and were finally stuck within the capillary bed of the tissue. During microsphere injection, a reference withdrawal with a constant rate of 5 mL/min was taken from a catheter placed in the descending thoracic aorta. Microspheres were retrieved postmortem by digestion of tissue or reference blood samples with 2 mol/L potassium hydroxide and subsequent filtration. The number of retrieved microspheres was estimated by measuring the respective dye content, and absolute tissue blood flow was calculated using the microsphere content in the reference withdrawal. The AAR within the two heart slices which contained the central AAR was cut into transmural segments of approximately 1 g each. Depending on the size of the respective slice and the size of the AAR, between two and four segments were retrieved from each slice. For calculation of regional myocardial blood flow within AAR_S_ and AAR_AFW_, respectively, only tissue samples which were completely allocated within the AAR_S_ or AAR_AFW_ were considered. Samples overlapping with AAR_IM_ were excluded. For that reason, the *n*‐values for data on regional myocardial blood flow are less than the total number of analyzed experiments. Regional myocardial blood flow is expressed in ml/min/g of tissue.

### Exclusion criteria

We did not define a priori exclusion criteria in this retrospective study. Experiments in which the protocol was not completed due to technical or surgical complications or with defibrillation‐resistant ventricular tachycardia/fibrillation were not considered for analysis. In addition, when TMBF within the total AAR was larger than 0.06 mL/min/g at 5 min after onset of ischemia, we assumed this to indicate insufficiently severe ischemia, and these experiments were also excluded. Given the above definition for segmentation of the AAR, only heart slices with a distinct right and left ventricular cavity were analyzed.

### Statistics

Data are given as mean ± SD. Statistics were performed with SigmaStat 3.5 (SigmaStat 3.5, Erkrath, Germany). Total AAR and IS within the total AAR, size of AAR_S_ and AAR_AFW_, IS within AAR_S_ and AAR_AFW_, regional myocardial blood flow, and systemic hemodynamics were analyzed by two‐way ANOVA with Fisher's least significant difference post‐hoc tests. ST‐segment elevation was analyzed by a mixed model analysis (SAS 9.4, SAS Institute Inc., Cary, NC) for the fixed effects “group,” “time,” and the interaction “group*time,” considering random effects induced by the individual animal. A *P* < 0.05 was considered significant.

## Results

From all experiments performed between November 2012 and November 2018 with one of the above described experimental protocols, 58 pigs were not included in the present analysis (technical or surgical problems *n* = 20; defibrillation‐resistant ventricular tachycardia/fibrillation *n* = 24; TMBF> 0.06 mL/min/g at 5 min ischemia *n* = 14). The incidence of ventricular fibrillation was 44% in I/R, 44% in IPC + I/R, 44% in I/R + POCO, 28% in RIPC + I/R, and 32% in I/R + RPER and not different between groups (*P* = 0.59). In pigs with ventricular fibrillation, it was terminated by 2.5 ± 2.4 defibrillations per pig.

Heart rate, LVP_max_, and dP/dt_max_ did not differ between groups (Table 1). As expected, LVP_max_ and dP/dt_max_ decreased after onset of ischemia and only partially recovered during reperfusion. Please note that data on ST‐segment elevation have been previously published and presented as figures (Amanakis et al. 2019). The time course of ST‐segment elevation differed between protocols (Table 2). ST‐segment elevation was negligible at baseline and not different between groups. With I/R, ST‐segment elevation was increased at 5 min ischemia and remained unchanged at 55 min ischemia. At 10 min reperfusion, ST‐segment elevation was increased further and then gradually recovered, but remained slightly above baseline at 120 min reperfusion. IPC + I/R and RIPC + I/R did not significantly attenuate ST‐segment elevation at 5 min ischemia (there was a trend for IPC + I/R), but did so at 55 min ischemia. This attenuation of ST‐segment elevation by IPC + I/R and RIPC + I/R remained apparent up to 30 min reperfusion. I/R + RPER attenuated ST‐segment elevation after the conditioning maneuver at 55 min ischemia and up to 10 min reperfusion. ST‐segment elevation with I/R + POCO was not different from that with I/R at baseline and during ischemia, but the further increase in ST‐segment elevation at 10 min reperfusion was abrogated.

**Table 1:** Systemic hemodynamics.

		HR [1/min]	LVP_max_ [mmHg]	dP/dt_max_ [mmHg/s]
I/R (*n* = 39)	Baseline	119 ± 16	90 ± 9	1767 ± 345
	I5	115 ± 16	80 ± 8*	1443 ± 263*
	I55	119 ± 16	82 ± 10*	1532 ± 390*
	R10	119 ± 15	81 ± 9*	1559 ± 423*
	R30	118 ± 14	81 ± 9*	1614 ± 437*
	R60	118 ± 13	80 ± 9*	1633 ± 340*
	R120	118 ± 14	78 ± 9*	1520 ± 342*
IPC + I/R (*n* = 16)	Baseline	112 ± 12	90 ± 10	1696 ± 344
	I5	105 ± 12*	82 ± 9*	1402 ± 262*
	I55	109 ± 12	81 ± 9*	1437 ± 315*
	R10	110 ± 14	83 ± 7*	1554 ± 364*
	R30	109 ± 14	80 ± 7*	1459 ± 361*
	R60	110 ± 14	80 ± 9*	1473 ± 371*
	R120	109 ± 15	78 ± 10*	1408 ± 419*
I/R + POCO (*n* = 18)	Baseline	121 ± 13	85 ± 9	1685 ± 415
	I5	117 ± 17	78 ± 6*	1384 ± 297*
	I55	119 ± 14	78 ± 7*	1485 ± 411*
	R10	117 ± 13	76 ± 7*	1482 ± 470*
	R30	118 ± 13	75 ± 7*	1583 ± 526
	R60	117 ± 15	73 ± 6*	1452 ± 354*
	R120	114 ± 13*	72 ± 6*	1325 ± 350*
RIPC + I/R (*n* = 31)	Baseline	111 ± 13	86 ± 7	1581 ± 435
	I5	107 ± 14	77 ± 6*	1259 ± 274*
	I55	111 ± 17	77 ± 9*	1317 ± 284*
	R10	113 ± 19	77 ± 10*	1377 ± 339*
	R30	115 ± 17	77 ± 9*	1436 ± 322*
	R60	115 ± 15	77 ± 9*	1449 ± 340*
	R120	113 ± 16	75 ± 10*	1337 ± 371*
I/R + RPER (*n* = 28)	Baseline	111 ± 12	89 ± 10	1827 ± 472
	I5	105 ± 12*	81 ± 10*	1366 ± 291*
	I55	105 ± 14*	76 ± 9*	1342 ± 299*
	R10	110 ± 12	77 ± 8*	1445 ± 401*
	R30	107 ± 11	77 ± 9*	1481 ± 427*
	R60	108 ± 13	79 ± 10*	1523 ± 436*
	R120	110 ± 13	77 ± 9*	1456 ± 428*

I/R, ischemia/reperfusion; IPC + I/R, local ischemic preconditioning; I/R + POCO, local ischemic postconditioning; RIPC + I/R, remote ischemic preconditioning; I/R + RPER, remote ischemic perconditioning; I5/55, 5/55 min ischemia; R10/30/60/120, 10/30/60/120 min reperfusion; HR, heart rate; LVP_max_, maximal left ventricular pressure; dP/dt_max_, maximal rate of rise of left ventricular pressure.

Data are mean ± SD; *P < 0.05 versus baseline; two‐way ANOVA for repeated measures and Fisher's least significant difference post‐hoc tests.

**Table 2:** ST‐segment elevation was different between the five protocols.

	ST‐segment elevation [µV]						
	Baseline	I5	I55	R10	R30	R60	R120
I/R (*n* = 29)	50 ± 30	271 ± 124	246 ± 150	323 ± 159	209 ± 120	132 ± 75	103 ± 59
IPC + I/R (*n* = 15)	50 ± 25	215 ± 61	152 ± 62*	204 ± 50*	138 ± 40*	93 ± 30	81 ± 21
I/R + PoCo (*n* = 9)	49 ± 36	245 ± 119	232 ± 92	242 ± 138*	164 ± 85	133 ± 60	92 ± 51
RIPC + I/R (*n* = 21)	69 ± 55	258 ± 88	186 ± 43*	255 ± 109*	184 ± 72	127 ± 59	114 ± 56
I/R + RPER (*n* = 18)	45 ± 46	283 ± 111	180 ± 52*	235 ± 131*	177 ± 94	103 ± 55	114 ± 56

I/R, ischemia/reperfusion; IPC + I/R, local ischemic preconditioning; I/R + POCO, local ischemic postconditioning; RIPC + I/R, remote ischemic preconditioning; I/R + RPER, remote ischemic perconditioning; I5/55, 5/55 min ischemia; R10/30/60/120, 10/30/60/120 min reperfusion.

These data have been previously published and presented as figures (Amanakis et al., 2019). Data are mean ± SD; *P < 0.05 versus I/R; mixed model analysis for the fixed effects “group,” “time,” and the interaction “group*time,” considering random effects induced by the individual animal.

**Figure 2:**
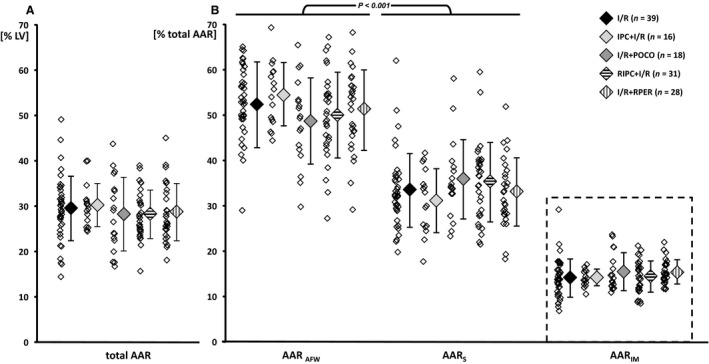
(A) Area at risk (AAR) expressed as fraction of the left ventricle, and (B) as fraction of the total AAR separated for myocardium allocated to the septum (AAR_S_) or the anterior free wall (AAR_AFW_). The intermediate segment of the AAR (AAR_IM_; dashed box) was not clearly allocated to either AAR_S_ or AAR_AFW_ and therefore excluded from statistical analyses. The part of the AAR allocated to AAR_AFW_ was larger than that allocated to AAR_S_. Data are given as individual data points and mean ± SD (two‐way ANOVA for repeated measures and Fisher's least significant difference post‐hoc tests). I/R, ischemia‐reperfusion; IPC, ischemic preconditioning; POCO, ischemic postconditioning; RIPC, remote ischemic preconditioning; RPER, remote ischemic preconditioning.

The total AAR, expressed as a fraction of the LV, was similar in all experiments without differences between protocols (Fig. 2A). About 15% of the AAR could not be unequivocally attributed to either the septum or the anterior free wall using the above described separation; this intermediate segment of the AAR was excluded from further analyses. The size of the AAR allocated to the septum was smaller than that allocated to the anterior free wall (*P* < 0.001) (Fig. 2B). In all groups, TMBF at baseline was similar and similarly reduced with ischemia, regardless of its allocation to either AAR_S_ or AAR_AFW_, respectively (Table 3).

**Table 3:** Transmural myocardial blood flow was not different between myocardium within the area at risk allocated either to the septum (AAR_S_) or the anterior free wall (AAR_AFW_), neither at baseline nor at 5 min ischemia.

Baseline [ml/min/g]		5 min ischemia [ml/min/g]	
AAR_S _(*n* = 43)	AAR_AFW _(*n* = 44)	AAR_S_ (*n* = 39)	AAR_AFW_ (*n* = 41)
0.73 ± 0.41	0.79 ± 0.44	0.030 ± 0.02*	0.032 ± 0.02*

Data are mean ± SD; *P < 0.001 versus baseline; two‐way ANOVA for repeated measures and Fisher's least significant difference post‐hoc tests.

**Figure 3:**
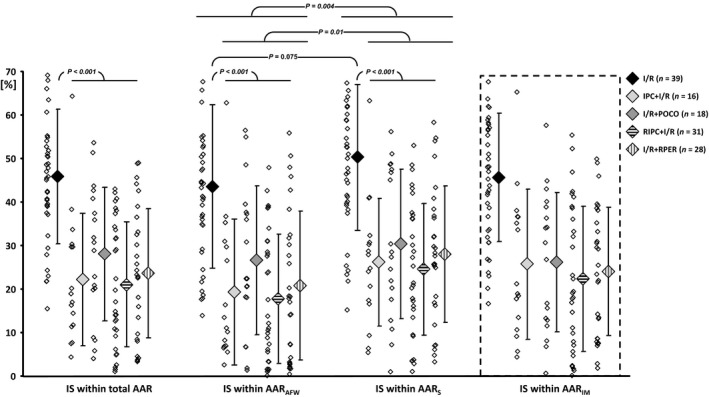
(A) Infarct size (IS), expressed as a fraction of the total area at risk (AAR) and (B) as fraction of the myocardium allocated to the septal part of the AAR (AAR_S_) and as fraction of the part of the AAR within the anterior free wall (AAR_AFW_). Myocardium within the intermediate part of the AAR (AAR_IM_; dashed box) was not clearly allocated to either AARS or AARAFW and therefore excluded from statistical analyses. Ischemic conditioning robustly reduced infarct size (*P* < 0.001 vs. I/R). Infarct size in the septal parts of the AAR was larger in both protected and unprotected myocardium. Data are given as individual data points and mean ± SD (two‐way ANOVA for repeated measures and Fisher's least significant difference post‐hoc tests). I/R, ischemia‐reperfusion; IPC, ischemic preconditioning; POCO, ischemic postconditioning; RIPC, remote ischemic preconditioning; RPER, remote ischemic preconditioning.

All four ischemic conditioning maneuvers reduced IS, without a notable difference between the respective maneuvers (Fig. 3A). When IS was calculated separately as fraction of the AAR_S_ or AAR_AFW_, respectively, IS was larger in the septum than in the anterior free wall. This effect was statistically significant for pooled data from pigs with ischemic conditioning and tended to be significant in pigs with I/R only (*P* = 0.075).

## Limitation of methods

The underlying data were retrospectively analyzed from experiments performed in a single institution over a period of 6 years. Such approach has been used by others before (Rossello et al. 2018) and is in line with the 3R (replace, reduce, refine) principle since all experiments have been performed and reported previously in another context. The algorithms for the cardioprotective maneuvers have been adapted from published maneuvers in clinical settings [IPC (Buyukates et al. 2005), RIPC (Munk et al. 2010), RPER (Bøtker et al. 2010), POCO (Staat et al. 2005)] with slight modifications. These maneuvers robustly reduced IS in our prior studies (Skyschally et al. 2013; Skyschally et al. 2015; Gent et al. 2017; Kleinbongard et al. 2018). We therefore did not modify the algorithms in number and duration of ischemia/reperfusion cycles to further optimize the magnitude of IS reduction (Johnsen et al. 2016). Anesthesia was maintained with isoflurane which might facilitate cardioprotection (Haroun‐Bizri et al. 2001). However, the anesthetic regimen was identical in all pigs. The translational value of experiments performed in young and healthy animals without any co‐medication is limited since these factors are potential confounders of cardioprotection by ischemic conditioning (Ferdinandy et al. 2014; Hausenloy et al. 2017) in patients at risk of acute myocardial infarction (Heusch and Rassaf, 2016; Heusch, 2017).

## Discussion

Confirming the data of Schulz et al. (2005), the IS within the LAD perfusion territory in the present study differed between the septal and anterior free wall perfusion territories. In both, our present and the Schulz et al. study, relative IS in control hearts without an ischemic conditioning maneuver was larger in the septum than in the anterior free wall, rendering the septal myocardium more sensitive to infarction than the myocardium in the free anterior wall. Given the more distal LAD occlusion site in our experiments than that in the Schulz et al. study, the total AAR was smaller than that in the study by Schulz et al. (2005), but the relative distribution of the AAR between the septum and the anterior free wall was similar.

We did not observe any difference in regional myocardial blood flow between septal areas and areas within the anterior free wall, neither at baseline nor during ischemia. This lack of difference precludes an association of regional differences in infarct development with regional differences in myocardial blood flow at baseline (Ghaleh et al. 1996) or during ischemia. However, an increased capillary‐to‐myocyte distance in septal myocardium, as reported by Schulz et al. (2005), might be a reasonable explanation for greater ischemic injury to septal myocardium.

Ischemic conditioning robustly reduced the IS. In the study by Schulz et al. local ischemic preconditioning abolished the difference in relative IS between the septum and the anterior free wall, whereas in our study, relative IS remained larger in the septum, notwithstanding profound reduction of IS by ischemic conditioning. Whether such difference in the magnitude of protection between septum and anterior free wall and between our present and the Schulz et al. study can be attributed to the different experimental models, that is, 90 min low‐flow ischemia versus 60 min coronary occlusion, remains open.

Despite some inherent differences between septal and anterior free wall myocardium, the magnitude of IS reduction by all tested ischemic conditioning maneuvers results in a similar pattern, regardless if analyzed within the total AAR or within septal or anterior free wall areas. There is no evidence that a regionally different myocardial innervation impacts on infarct development and protection from it. Lieder et al. have recently described the involvement of vagal pathways in IS reduction by remote ischemic conditioning using the same experimental model as in the present analysis; bilateral vagal dissection abolished cardioprotection by remote ischemic conditioning (Lieder et al. 2018). The present study does not suggest that the vagal innervation of the septum and the anterior free wall is different. Whether the chamber‐specific difference in right and left heart reactive oxygen species handling (Schlüter et al. 2018) relates also to septal myocardium, and thus increases its susceptibility for infarction, remains unclear. It is also unclear whether or not the differences in IS between septum and anterior free wall can be extrapolated to coronary microvascular obstruction (Heusch, 2016; Heusch et al. 2019). A future comprehensive study might include simultaneously derived data on all potentially influencing factors such as regional myocardial load (estimated by hemodynamics and echocardiography), regional myocardial blood flow at multiple time points during ischemia and reperfusion, and regional structural differences of the myocardium (estimated by histology targeting cardiomyocytes, microvasculature, and connective tissue). Whether or not differences in intracellular signal transduction may also impact on susceptibility for infarction is unknown.

In summary, IS is larger in the septum than in the anterior free wall, but ischemic conditioning maneuvers are effective in the entire LAD perfusion territory. We can only speculate that our findings can be extrapolated to the left circumflex coronary artery and right coronary artery perfusion territories.

## Conflict of interest

The authors declare that they have no conflict of interest.

## Funding information

The present study was supported by the German Research Foundation (SFB 1116 B08).

## Ethical approval

All applicable international, national, and/or institutional guidelines for the care and use of animals were followed.
